# UCbase 2.0: ultraconserved sequences database (2014 update)

**DOI:** 10.1093/database/bau062

**Published:** 2014-06-19

**Authors:** Vincenzo Lomonaco, Riccardo Martoglia, Federica Mandreoli, Laura Anderlucci, Warren Emmett, Silvio Bicciato, Cristian Taccioli

**Affiliations:** ^1^Computer Engineering Department, University of Modena, Via Campi 213/b, 44100, Modena, ^2^Department of Statistical Sciences, University of Bologna, Via Belle Arti 41, 40126, Bologna, Italy, ^3^Department of Genetics, Environment and Evolution, Genetics Institute, University College London, London, WC1E 6BT, UK and ^4^Department of Life Sciences, Center for Genome Research, University of Modena and Reggio Emilia, Via G. Campi 287, 41100, Modena, Italy

## Abstract

UCbase 2.0 (http://ucbase.unimore.it) is an update, extension and evolution of UCbase, a Web tool dedicated to the analysis of ultraconserved sequences (UCRs). UCRs are 481 sequences >200 bases sharing 100% identity among human, mouse and rat genomes. They are frequently located in genomic regions known to be involved in cancer or differentially expressed in human leukemias and carcinomas. UCbase 2.0 is a platform-independent Web resource that includes the updated version of the human genome annotation (hg19), information linking disorders to chromosomal coordinates based on the Systematized Nomenclature of Medicine classification, a query tool to search for Single Nucleotide Polymorphisms (SNPs) and a new text box to directly interrogate the database using a MySQL interface. To facilitate the interactive visual interpretation of UCR chromosomal positioning, UCbase 2.0 now includes a graph visualization interface directly linked to UCSC genome browser.

**Database URL:** http://ucbase.unimore.it

## Introduction

Ultraconserved sequences (UCRs) are genomic sequences that were found identical comparing human, rat and mouse genomes (1). Because of their extreme conservation it has been postulated that these regions must have biological functions essential to mammal cells (2). Although the biological function of the majority of UCRs is still unknown, few ultraconserved regions have been functionally implicated in transcriptional enhancement, alternative splicing or nonsense mediated decay mechanisms (3–5). UCRs may also exert their function as noncoding RNAs that regulate other RNAs (6) or may participate in chromatin regulation (7). Moreover, several studies demonstrated that expression levels of UCR-derived transcripts are deregulated in human cancer tissues (6, 8, 9) and that some UCRs undergo CpG island hypermethylation-associated silencing (10).

Here, we present UCbase 2.0, an updated version of UCbase (11), a comprehensive resource for the analysis of genomic regions that are 100% conserved in human, mouse and rat genomes. As compared with the previous release, UCbase 2.0 has much wider database content, a completely newly redesigned user interface and novel software architecture. Instead, information about microRNAs (miRNAs) has been removed because of the availability of more specific Web resources dedicated to miRNA analysis.

## Database content update

UCbase 2.0 uses chromosomal coordinates from the latest version of the human genome assembly (hg19/GRCh37) and all UCRs are linked to the UCSC genome browser (http://genome.ucsc.edu/), thus allowing researchers to visualize specific UCRs within the respective genes and chromosomes. UCbase 2.0 is now maintained on an Apache Web Linux 64 processor server hosted by the bioinformatics facility of the University of Modena and Reggio Emilia Center for Genome Research (www.cgr.unimore.it).

### Database architecture and data acquisition

The database architecture has been redesigned to integrate all needed information about UCRs in a complete, simple-to-understand and consistent Web interface solution. UCbase 2.0 includes:

– ‘Ultraconserved sequences’ together with their genomic information (identification code and chromosome coordinates);
– ‘Gene names’ containing the UCRs and their information (gene symbol, chromosome coordinates, etc.);
– ‘Pathology names’ correlated to a particular gene, with Mendelian Inheritance in Man (MIM) (12) description and name, including a complete hierarchy explicating the generalization properties between them (subtypes) and a series of hyperlinks to correlated entries in popular and renowned thesauri;
– ‘SNPs’ located within a specific UCR, with information about polymorphism id, gene id and chromosome coordinates, as well as SNPs located up- and downstream (500 bp) a single UCR;
– ‘Splicing event types’ correlated to a given gene and their information (chromosomal coordinates, description, etc.).

**Figure 1.**
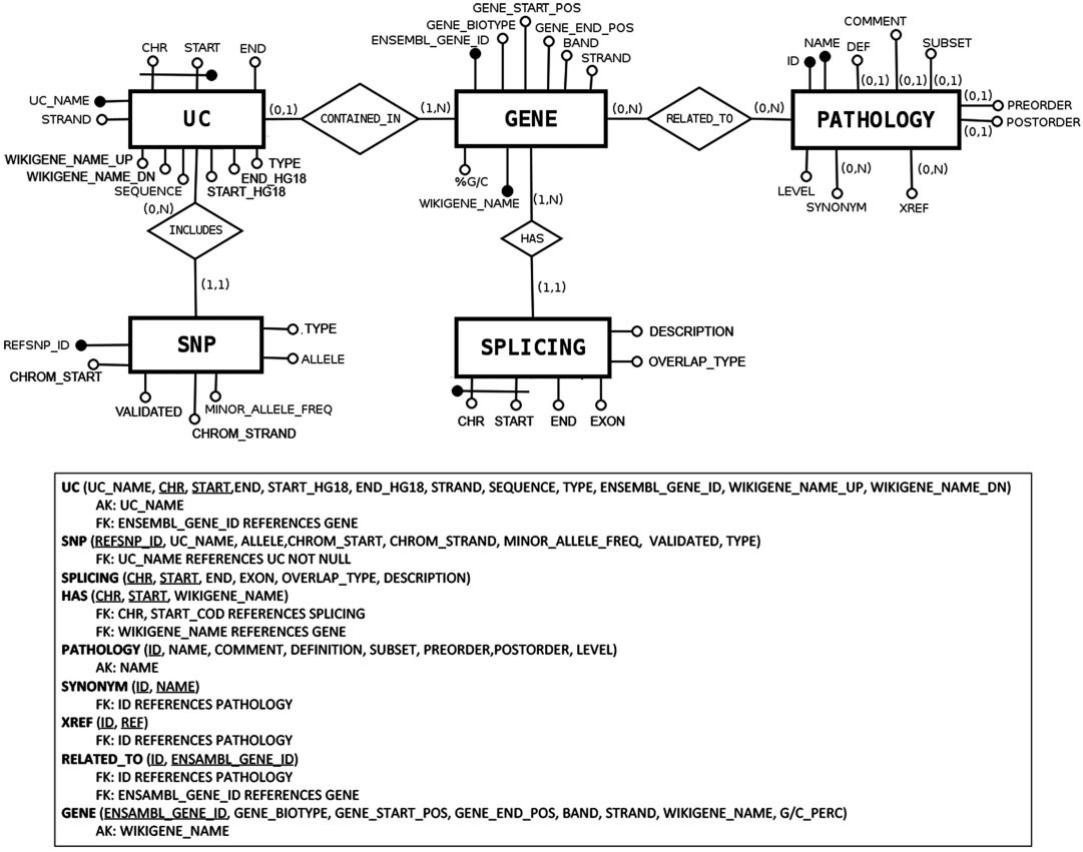
Database structure described in entity–relationship model (ER model) standard language.

The architecture of UCbase 2.0 is depicted in Figure 1, which shows the logical schema of the database. UCbase 2.0 is automatically and periodically populated by a Web extraction software specifically designed to implement this updating step. Specifically, all raw data and updated chromosomal coordinates are extracted from the BioMart portal (13) through automated Java scripts invoking the relevant Web service through a Simple Object Access Protocol (SOAP) interface. Information about the pathology hierarchy and hyperlinks has been derived from the complete Human Disease Ontology available on the ‘Open Biological and Biomedical Ontologies’ portal (14). UCbase 2.0 adopts the standardized Systematized Nomenclature of Medicine Clinical Terms (SNOMED CT) for disorder nomenclature (15). SNOMED CT is a systematic computer-processable collection of medical terms, in human and veterinary medicine, which provides codes, terms, synonyms and definitions covering anatomy and diseases. SNOMED CT allows adopting a consistent approach to index, store, retrieve and aggregate medical data across specialties and sites of care.

### Web user interface and query types

**Figure 2.**
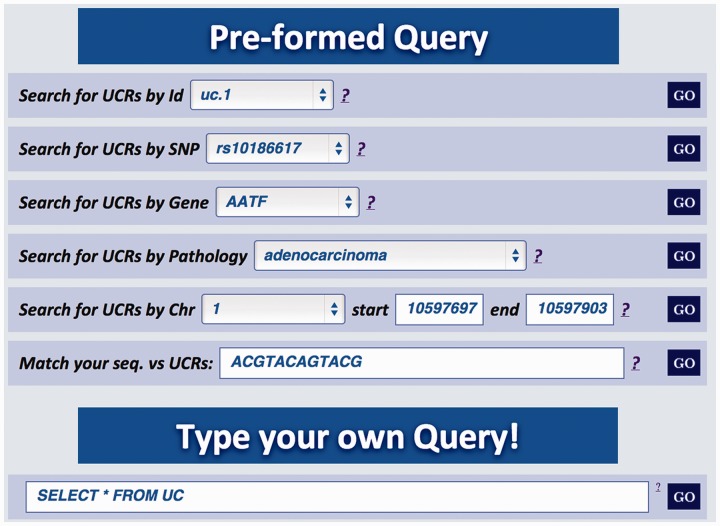
Multiqueries text box. Multiple queries can be performed typing the names of selected UCR SNP, gene, pathology, genomic location or a specific nucleotide sequence. It is also possible to directly interrogate UCbase using MySQL script language.

Major improvements of the Web user interface are aimed at facilitating the extraction of relevant information about genes in which the UCRs are located. For instance, in this updated version it is possible to investigate if genes containing UCRs have SNPs or undergo splicing events. As in the previous version, the UCbase 2.0 interface still contains prestructured queries (‘Preformed Query’) but, in addition, now it includes a text box to directly interrogate the database using SQL commands (‘Type your own Query’ input field, Figure 2):

#### Preformed queries

**Figure 3.**
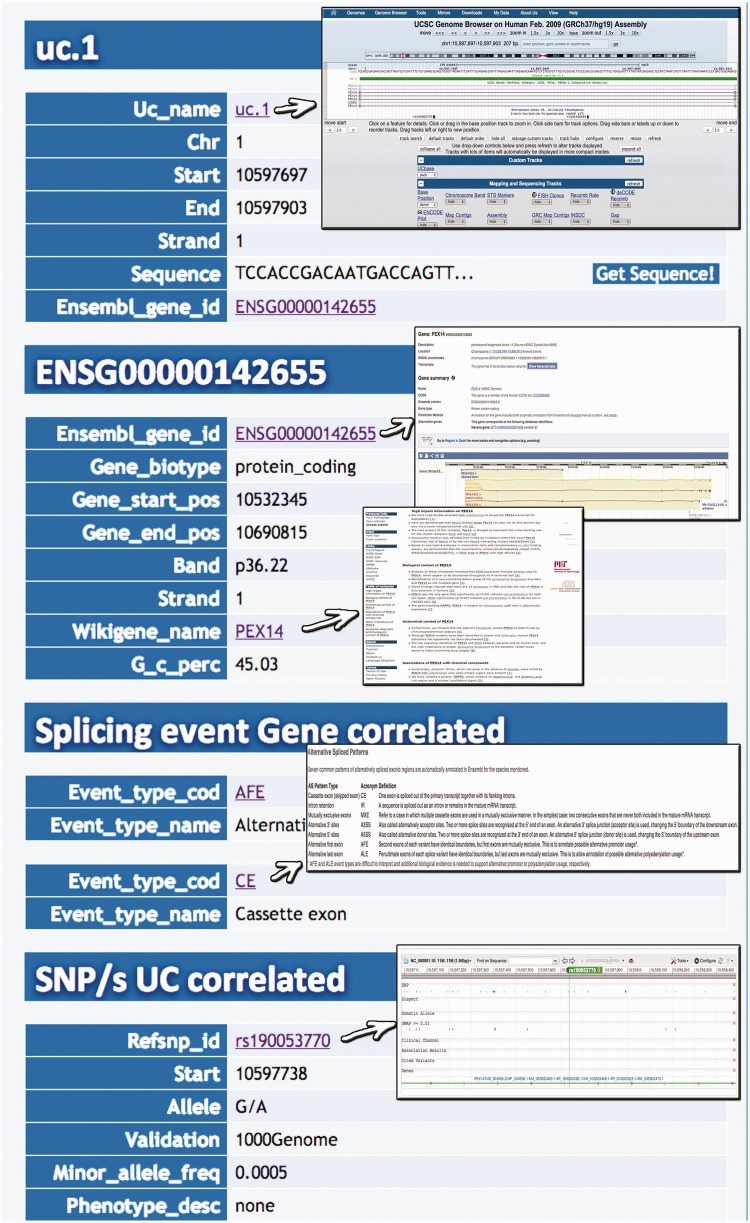
Result for UCR Id query. Typing the Id name of a particular UCR (uc.1 in this case) it is possible to retrieve information about chromosome coordinates, the gene in which the UCR is located, the gene splicing events and the SNPs located in that particular UCR.

**Figure 4.**
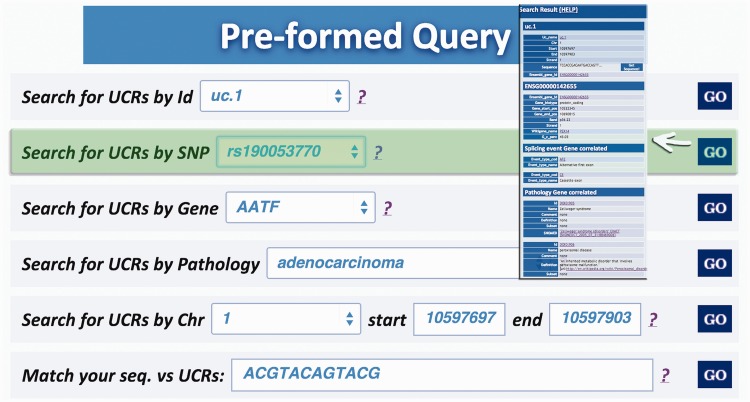
Query result for SNP search. This result shows the UCR in which that particular SNP is located (in this case rs190 053 770) together with chromosomal coordinates, allelic frequency, validation and phenotype information.

**Figure 5.**
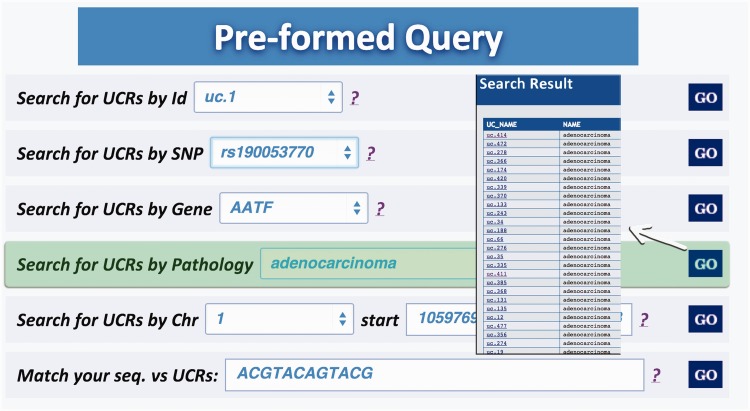
Result for Pathology query. This output shows the UCRs involved in a particular pathology correlated to the genes in which the UCRs are located.

**Figure 6.**
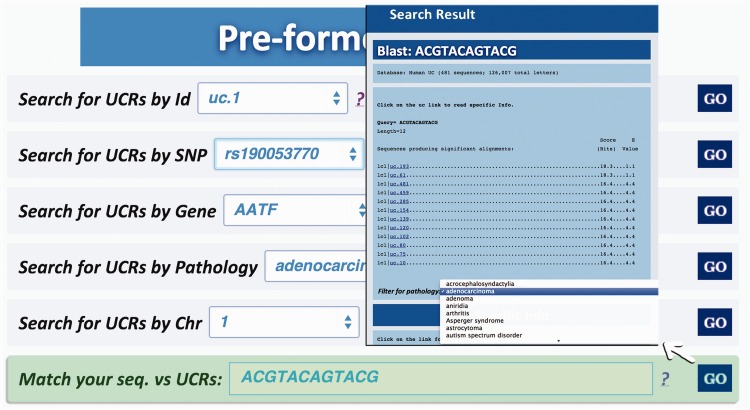
Result for BLAST search. This page shows the output of a sequence typed by the user (in this case ACGTACAGTACG), which matches with several ultraconserved elements. It is also possible to filter for those UCAs showed in the output, which are located in genes involved in specific pathologies.

UCbase 2.0 offers six different prestructured queries to interrogate UCR sequences and related information. Specifically:

Query type 1: searches UCRs (and all their related information) using the UCR Id (Figure 3). With this type of query, it is also possible to select multiple ids to simultaneously retrieve information about multiple UCRs;
Query type 2: retrieves UCRs containing a specific SNP using dbSNP Ids (http://www.ncbi.nlm.nih.gov/SNP/) (16) (Figure 4);
Query type 3: searches UCRs correlated to a specific gene;
Query type 4: retrieves all UCRs contained in a given chromosomal location identified by chromosome number and start and end chromosomal coordinates;
Query type 5: retrieves all UCRs correlated to a given pathology (and all its subtypes) (Figure 5);
Query type 6: searches all UCRs (and their parts) approximately matching a given sequence using BLAST (17). The returned UCRs are ranked by matching score (E-value) and can be subsequently filtered by a given pathology (and all its subtypes) (Figure 6).

A search starting from the UCR Id (‘query type 1’) returns the link to UCSC genome browser (Figure 3), the gene/region in which the UCR is located and the genes located up/downstream that UCR (Wikigene_name_up/dn), the chromosome region in which the UCR is located (in both hg18 and hg19 genome references), the splicing events and pathology related to that specific gene (Figure 2). Furthermore, both searching UCRs using UCR Ids or performing a ‘query type 2’ returns the SNPs located within a specific UCR and those located 500 bp up- and downstream the same ultraconserved sequence together with chromosomal coordinates, allelic frequency, validation and phenotype information (Figure 4). Searching for gene or chromosome coordinates (‘query types 3 and 4’, respectively) results all the information related to the UCRs located in that specific genomic region, whereas ‘query type 5’ outputs a table containing all pathologies related to the gene or chromosome region in which a particular UCR is located (Figure 5). When directly searching for genes and SNPs, UCbase 2.0 shows only the genomic features that overlap UCRs. This has been made to avoid confusion and keep the tables clearer and more readable. The new BLAST-based search query instead allows matching a sequence against the entire UCR sequence database (‘query type 6’) and optionally provides the new opportunity to filter results for the UCRs located in genes involved in specific pathologies (Figure 6). In particular, ‘query type 6’ is solved through approximate matching using NCBI BLASTN (http://www.ncbi.nlm.nih.gov/gene). To this end, we embedded in UCBase 2.0 a database of all UCR sequences in FASTA format, which is used to match the submitted request through the BLASTN command. Finally, when a ‘query of type 6’ is submitted, BLASTN results can be filtered to show only those sequences related to the specified pathology and its subtypes.

#### Queries using the SQL command line

In addition to the six different prestructured queries, UCbase 2.0 can be directly interrogated through custom-defined SQL code chunks using an *ad* *hoc* command line (see Figure 2, ‘Type your own Query’ text box). For instance, the user can perform simple queries such as ‘Return the number of UCRs currently in the database’:

SELECT COUNT(*) FROM UC

or more complex queries as ‘List all genes correlated to UCRs, ranked by the number of UCRs they contain from high to low’:

SELECT ENSEMBL_GENE_ID, COUNT(*) FROM UC GROUP BY ENSEMBL_GENE_ID ORDER BY 2 DESC

Moreover, even though ‘Queries types 1 to type 6’ are solved through the vanilla SQL (18) queries issued to the MySQL DBMS (http://dev.mysql.com/), the same request can be directly performed using the SQL command line. For example, to get the UCRs related to a given gene (‘query type 3’, e.g. ‘AATF’), the query syntax will be:

SELECT UC_NAME, SEQUENCE FROM UC, GENE WHERE UC. ENSEMBL_GENE_ID = GENE.ENSEMBL_GENE_ID AND GENE.WIKIGENE_NAME = ‘AATF’

In general, through the command line, the user can construct any type of query combining all fields and attributes comprised in the database structure (see Figure 1). As an example, it is possible to retrieve all the UCRs located within long noncoding RNAs just imputing this SQL code:

SELECT UC_NAME FROM UC, GENE WHERE UC. ENSEMBL_ GENE_ID = GENE.ENSEMBL_GENE_ID AND GENE_BIOTYPE=‘lincRNA’

It is also possible to extract the information included in any specific field of a table typing this query:

SELECT DISTINCT *field* FROM table

For example, to retrieve all the GENE_BIOTYPE features (miRNA, lincRNA, antisense, etc.) from the GENE table, the query is:

SELECT DISTINCT GENE_BIOTYPE FROM GENE

## Discussion

Several other resources are currently available for the analysis of UCRs, e.g. UCNEbase (19), cneViewer (20), CONDOR (21), VISTA Enhancer browser (22), Ancora (23), ECR browser (24), TFCONES (25), FANTOM 5 enhancer atlas (26) and FANTOM 5 promoter atlas (27). In detail:

– UCNEbase provides information on conserved regions focusing on their evolutionary relationships in >18 vertebrates. Specifically, UCNEbase introduces a coherent nomenclature for ultraconserved noncoding elements reflecting their respective associations with likely target genes and is particularly useful to any computational or evolutionary biologist interested in conserved noncoding DNA elements in vertebrates. As UCNEbase relies on the UCSC genome browser to visualize UCRs and their related characteristics, a large part of query results is returned as UCSC genome browser custom tracks and requires the downstream customization of the UCSC browser to display all UCR characteristics (as SNPs or spicing events);
– cneViewer is a database of conserved sequences between human and zebrafish genomes;
– CONDOR and VISTA enhancer browser consist of experimental annotation of noncoding elements based on *in vivo* reporter gene assays in zebrafish and mouse;
– Ancora and ECR browser offer data for a comparable number of species, restricting some existing resources to selected genomic regions;
– TFCONES provides conservation information for human, mouse and fugu genomes;
– FANTOM 5 enhancer and promoter atlas provide, through Cap Analysis of Gene Expression (CAGE) technology (28), comprehensive expression profiles and functional annotation of mammalian cell-type-specific enhancer and promoter regions enabling gene regulatory network detection not only limited to UCRs.

UCbase 2.0 represents a completely distinct application with significantly different characteristics and scopes. Specifically, UCbase 2.0 focuses on UCRs as published by Bejerano *et al.* in 2004 (1) and represents the sole database directly linking UCRs to genes and/or regions involved in genetic or nongenetic disorders (11), giving, at the same time, the opportunity to retrieve information about the genomic regions in which the UCRs are located (genes, SNPs, splicing events, etc.). UCbase 2.0 can be directly used through the database interface without the need to invoke and customize UCSC genome browser tracks. We believe that this characteristic makes UCbase 2.0 an easy-to-use tool for all users, limiting the need to access external resources (as the UCSC browser tracks) while preserving the possibility to perform exhaustive queries. Nevertheless, UCbase 2.0 data can also be preloaded to the UCSC genome browser through hyperlinks, thus allowing researchers with bioinformatics skills to explore its content in a more advanced manner. Finally, as far as we know, UCbase 2.0 is the sole tool that allows retrieving UCRs directly querying for a disease. Indeed, UCNEbase and other tools emphasize more on evolutionary and conservation aspects, whereas UCbase 2 is specifically designed to retrieve genomic information about sequences that are highly conserved between human, mouse and rat when related to diseases.

## Conclusion

UCbase 2.0 is the sole database containing the long 481 UCRs discovered in the genomes of human, mouse and rat by Bejerano *et al.* and identified as deregulated in cancer. The goal of this Web resource is to offer to researchers the opportunity to retrieve genomic information about a specific UCR and an advanced set of tools that correlate UCRs to disorders related to the genes containing ultraconserved regions. UCbase version 2.0 includes completely redesigned database architecture and query methods and a new user interface to efficiently combine results from different sources and locate genomic regions on UCSC genome browser. The system is supplemented with a new tool to directly interrogate the database through SQL commands. This feature enhances the output retrieval and is especially useful when multiple queries are submitted simultaneously to obtain complex results. Additionally, UCbase 2.0 automatically updates content information, such as human, mouse and rat UCRs and genomes from BioMart, using Java scripts. Although other alternatives are available to retrieve UCRs nomenclature, sequence data and annotation, UCbase 2.0 comprises a unique combination of features that allow biologists to analyze and discover relationships between UCRs and pathologies related to their genomic location. UCbase 2.0 relies on its own interface to retrieve UCRs information but it is highly interoperable with the UCSC genome browser showing UCR chromosome coordinates in custom tracks that are automatically preloaded to the UCSC browser through hyperlinks.

## Availability

The Web interface of UCbase 2.0 is freely available to academic users at http://ucbase.unimore.it. The database content formatted in tab-delimited, SQL and FASTA format is available for download at http://www.dsb.unimo.it/UCbase/downloads. A detailed manual with information about the Web service access is available at http://www.dsb.unimo.it/UCbase/help/help.pdf.

## Funding

This work was supported by Italian Ministry of University and Research (FIRB grant RBAP11T3WB) and by AIRC Special Program Molecular Clinical Oncology ‘5 per mille’ grant.

*Conflict of interest*. None declared.
